# Cutaneous Events Associated with Immunotherapy of Melanoma: A Review

**DOI:** 10.3390/jcm10143047

**Published:** 2021-07-08

**Authors:** Lorenza Burzi, Aurora Maria Alessandrini, Pietro Quaglino, Bianca Maria Piraccini, Emi Dika, Simone Ribero

**Affiliations:** 1 Department of Medical Sciences, Dermatology Clinic, University of Turin, 10126 Turin, Italy; lorenza.burzi@unito.it (L.B.); pietro.quaglino@unito.it (P.Q.); 2 Dermatology, Department of Experimental Diagnostic and Specialty Medicine (DIMES), University of Bologna, 40138 Bologna, Italy; aurora.alessandrini3@unibo.it (A.M.A.); biancamaria.piraccini@unibo.it (B.M.P.); emi.dika3@unibo.it (E.D.); 3 Dermatology, IRCCS Sant’Orsola Hospital, 40138 Bologna, Italy

**Keywords:** melanoma, immunotherapy, pigmentation disorders, vitiligo, melanosis, halo nevus, alopecia, poliosis

## Abstract

Immunotherapy with checkpoint inhibitors significantly improves the outcome for stage III and IV melanoma. Cutaneous adverse events during treatment are often reported. We herein aim to review the principal pigmentation changes induced by immune check-point inhibitors: the appearance of vitiligo, the Sutton phenomenon, melanosis and hair and nail toxicities.

## 1. Introduction

The function of the immune system in melanoma disease course is well established. Immune checkpoint inhibitors have shown promise in enhancing the immune system to fight against cancer cells and in providing a higher response rates than chemotherapies used in the past [1,2].

Tumor cells inactivate the process of immunosurveillance by expressing ligands of immune checkpoint pathways. Immune checkpoint inhibitors (ICI) are monoclonal antibodies which target two immune checkpoint pathways: (1) the cytotoxic T-lymphocyte antigen-4 (CTLA-4) that is expressed by activated T cells and transmits an inhibitory signal to T cells activation during immune response, and (2) programmed death-1 protein (PD-1) that promotes apoptosis of T cells in lymph nodes and reduces apoptosis in regulatory T cells.

This activation of T cells may also be responsible for the occurrence of immune-related adverse effects (AE) affecting different organs and systems; in particular, skin-related AE are reported in up to 30–50% of patients treated with ICIs [3]. Herein, we review the principal pigmentation changes induced by ICI: the appearance of vitiligo, the Sutton phenomenon, the melanosis and hair and nail toxicities.

## 2. Vitiligo

**Figure 1:**
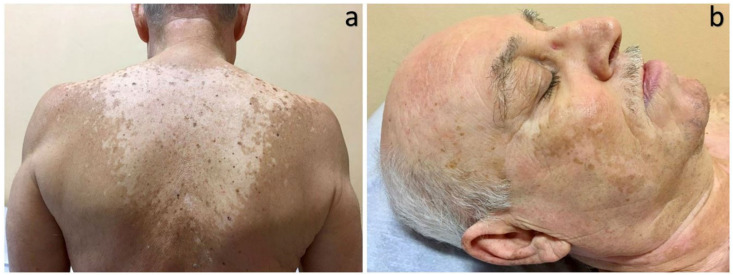
(**a**,**b**) ICI-induced vitiligo on photoexposed areas: several white dotted macules progressing toward larger patches.

Vitiligo is a skin pigmentation disorder characterized by the appearance of achromatic patches on the skin due to loss of melanocytes. Macules may present with a localized or generalized distribution pattern and may evolve into wide, depigmented areas. ICI-induced vitiligo is clinically different from “classic” vitiligo: patients who show vitiligo during anti-PD1 treatment usually develop white patches on areas with evident signs of chronic photoexposure with a typical pattern of depigmentation composed of several dotted macules evolving toward larger patches (Figure 1). In the majority of cases, personal or family history of autoimmune disorders including vitiligo or thyroiditis is negative. Moreover, the pattern of depigmentation in vitiligo-like lesions associated with immunotherapy does not involve the Koebner phenomenon, in contrast to what happens in vitiligo not related to anti-PD1, where the lesions are often induced by repetitive friction and usually involve periocular, perioral or wrists areas [4].

The appearance of vitiligo is one of the immune-related adverse effect in patients treated with immune checkpoint inhibitors and can affect up to 2–25% of melanoma patients receiving ICI-therapy, thus showing a higher prevalence than the one of melanoma patients not receiving ICI-therapy [5,6].

The cause of melanoma-associated vitiligo is not well known, but an autoimmune disorder has been hypothesized. The main effectors of this autoimmune mechanism are the cytotoxic CD8+ T cells that target melanocyte antigens, such as gp100, MART1, tyrosinase or tyrosinase-related proteins, responsible for melanin synthesis. CD8 T cells clone infiltrates both the tumor and vitiligo patches [7].

In 20.5% of all melanoma-associated vitiligo patients, the depigmentation occurs before the diagnosis of melanoma and estimated prevalence of this precocious type of melanoma-associated vitiligo is 0.15% [8].

Teulings et al. [9] have conducted a systematic review of 137 studies including stage III–IV patients affected by melanoma and treated with various immunotherapies, reporting that the overall cumulative incidence of vitiligo was 3.4% (2.0–6.3%).

Ipilimumab is a CTLA-4 inhibitor approved as treatment of metastatic melanoma. The overall incidence of ipilimumab-induced vitiligo is 2.0–14.3% [10,11], the depigmentation usually persists after treatment cessation, suggesting the correlation with the establishment of a lasting immune response [10].

Among the anti-PD1 agents currently used as treatments for melanoma, nivolumab and pembrolizumab are counted. Melanoma-associated vitiligo shows a higher rate of incidence related to anti-PD-1 therapy than to CTLA-4 inhibition [12,13].

The incidence of pembrolizumab related vitiligo ranges between 9.6% and 25% [14,15]. Vitiligo mainly presents in the generalized form (82% of cases), is localized in a few cases (12%) and rarely is a mixed form (1%) [14].

During treatment with nivolumab, the reported incidence of vitiligo is around 7.5% [12], but a higher rate of incidence was reported by Nakamura et al. [15], who observed skin depigmentation in 25.7% of their patients. Clinically, it mainly occurs with a localized pattern of distribution, affecting less than 10% of the body surface area [15].

Babai et al. [16] have reported depigmentation affecting predominantly photo-exposed body areas such as the face and the hands in 46% of cases of vitiligo induced by ICIs.

The combination therapy with the administration of both nivolumab and ipilimumab was associated with a shorter period to vitiligo onset compared to nivolumab in monotherapy (3.2 vs. 10.3 months) [17], and an increased rate of toxic cutaneous events was also documented with the combination therapy in some cases [18]. A phase 3 trial (CheckMate 067) compared nivolumab plus ipilimumab or nivolumab alone versus ipilimumab alone in advanced melanoma and reported the incidence of vitiligo in 9% in patients treated with combo-immunotherapies, in 10% of those who received nivolumab alone and in 5% of patients receiving ipilimumab alone, with a 4-year follow-up period [19].

Immune-related cutaneous adverse effects are generally correlated with improved progression-free survival and overall survival [14,15,20]. The response rate was higher in patients who developed vitiligo during treatment, and its onset within the first 5 months of immunotherapy was a positive indicator of treatment response [15].

In a cohort of 2954 patients, vitiligo was found to be an independent positive prognostic factor associated with overall survival in patients with stages III and IV melanoma and with distant metastasis-free survival in patients with stage III melanoma [8].

The appearance of vitiligo was not confirmed to be a significant factor in terms of overall survival when compared with the onset of other ICI-related AEs; meanwhile, a significant increase in survival rates was observed for patients who developed vitiligo during immunotherapy compared to those who did not develop any ICI-induced AEs [21].

Melanoma-associated vitiligo persists beyond completion of immunotherapy in melanoma patients [6,10] and may be a key factor in maintaining a long-lasting anti-tumoral immune response [22].

Other treatments other than ICI were reported to induce vitiligo-like depigmentation, suggesting an immunity response activation in targeted therapy [23]. In fact, the immune response is positively affected by BRAF inhibitors as previously described [24].

A recent study reported the repigmentation after the cessation of immunotherapy in six patients, which the authors suggest to be associated with melanoma progression [16].

The evidence of repigmentation heralding disease progression or recurrence has been supported by several other reports [25,26] and agreed that vitiligo is a sign of the immune system activity against melanoma cells with favorable impact on the prognosis.

Considering the positive impact of ICI-associated vitiligo on overall survival in melanoma patients, treatment should not be interrupted because of its appearance. Regarding its management, local or systemic immunosuppressive therapy, which are the first line of treatment for vitiligo in general practice, are not recommended in ICI-induced vitiligo because they can decrease ICI therapy response [27].

## 3. Halo Nevus

**Figure 2:**
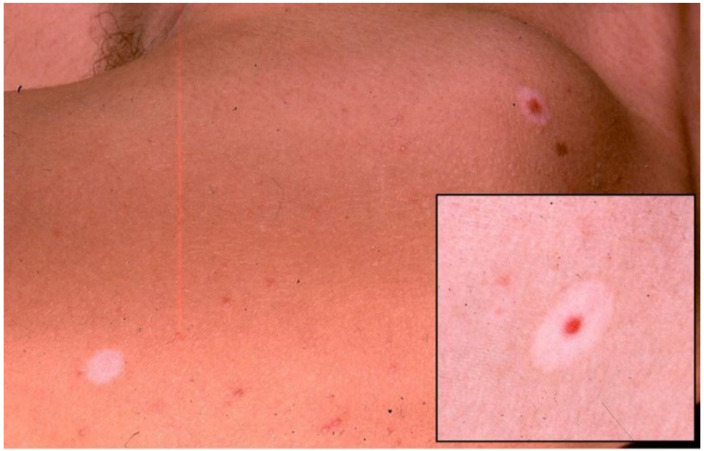
Halo nevus on lower abdomen and a white macule on the left as final ending of the regression phenomenon; in the magnification in the lower right portion of the image, the depigmented area surrounding the mole is more evident.

Sutton’s nevus (halo nevus) is a mole surrounded by an area of depigmentation that resembles a halo, resulting in spontaneous regression of the nevus (Figure 2).

The regression of a melanocytic lesion such as what happens in Sutton’s phenomenon must be differentiated from the regression that could appear in melanoma [28]. From a histological point of view, Sutton nevus is characterized by the presence of cells with rare mitosis and an abundant lymphocytic infiltration within the lesion, while the regressing melanoma presented with many immature and mitotic cells and an inflammatory infiltrate concentrated at the periphery. A prevalence of CD8+ over CD4+ cells was observed in halo nevus, in contrast to what was found in a regressing melanoma [29]. Halo phenomenon is not associated with fibrosis. This difference with respect to regressing melanoma was attributed to the different cytokine microenvironment; in fact, fibrogenic cytokines such as IL-6, TGF-β, TNF-α, bFGF and PDGF are expressed by melanoma while TNF-α, an antifibrogenic cytokine, was found to be more expressed in the halo nevus [29].

In literature, halo phenomenon is reported to appear after surgical removal of the primary melanoma lesion [20] or around cutaneous metastases and scars in melanoma patients [8]. Recently, halo nevus has also been reported as a clinical sign of melanoma disease progression [30]: in a cohort of sixteen people who presented with eruptive multiple halo naevi and underwent sequential PET scans over a 6-year period of follow-up, one patient was diagnosed of pulmonary metastases from a thin melanoma excised years previously, three patients had primary cutaneous melanoma and one had melanoma metastasis with unknown primary.

Immunotherapy-induced halo nevus reaction is reported to occur less frequently than vitiligo. Similar to what happen in vitiligo, the disappearance of pigmented lesions may be related to the onset of an immune response towards antigens shared by melanocytes and melanoma cells [31].

Few cases are reported in literature about the concomitant regression of multiple nevi and the maintained complete remission of metastatic melanoma during and after treatment by ipilimumab [32,33]. A stage IV melanoma patient with pulmonary and cerebral metastasis was treated with 3 mg/kg in four monthly injections and showed regression signs in cutaneous melanocytic naevi in concomitance with the regression of lung metastases after the fourth injection [32]. Similarly, another patient with diagnosis of stage IV melanoma who was administered four infusions of ipilimumab achieved a complete remission after 6 months. Simultaneously, he presented a generalized halo nevus phenomenon associated with a vitiligo patches localized on his hands and inguinal folds; the regression of his naevi appeared within 2 years, a total of 3 years after ipilimumab therapy [33].

Lastly, one case of halo nevi has been described in a patient with non-small cell lung cancer treated with atezolizumab, a programmed cell death ligand (PD-L1) antibody after 6 weeks of treatment, even if its prognostic significance in cancers other than melanoma remains to be evaluated [34].

## 4. Melanosis

Tumoral melanosis represents a rare clinico-pathologic feature which can be found in sites of completely regressed melanocytic lesions, including melanoma.

Tumoral melanosis usually presents as black or blue macules, papules or nodules clinically resembling a melanoma or cutaneous metastases. Because of this similarity, skin biopsy is essential for the diagnosis [35]. Histopathology of tumor melanosis demonstrates a large collection of melanophages and melanin pigment in subcutaneous tissue or dermis associated with the lack of melanocytes. Other typical histological features of tumoral melanosis are regression and fibrosis, lymphocytic infiltration and in some cases, the anamnestic data of the presence of a previous melanocytic lesion [36].

Cases of tumoral melanosis related to immunotherapy with ipilimumab, pembrolizumab and nivolumab have been reported. The most likely mechanism of regression that leads to tumoral melanosis seems to be the binding of T cells to melanoma tumor antigens, causing the influx of macrophages and the subsequent tumor demolition [37].

In the majority of cases reported so far, tumoral melanosis was found in proximity to the primary melanoma or in transit metastases [36,38], although three patients presented with tumoral melanosis at a distant site from the primary melanoma [35].

Recently, Jurgens et al. [36] reported the occurrence of melanosis in a cohort of 10 people affected by melanoma and treated with immunotherapy, with a period of latency between the starting of the treatment and the appearance of the melanosis ranging from 2 to 20 months. Immunotherapy with pembrolizumab was administered in four patients and ipilimumab in two patients, combo-immunotherapy with both ipilimumab and pembrolizumab was administered in three people, while one was treated with nivolumab.

Staser et al. [39] described a case of tumoral melanosis that arose close to a previously excised melanoma in a patient presenting concomitant in-transit metastases as a sign of recurrence during ipilimumab treatment with a short latency (2 months).

A few more clinical reports described the appearance of tumoral melanosis during treatment with pembrolizumab [40,41,42,43] after a period of therapy from 2 to 9 months.

Prognosis of tumoral melanosis is inconstant, depending on the status of the primary tumor; poor prognosis is reported for patients with metastatic disease while disease-free longer-term follow up usually affects those subjects with in-transit metastasis or in complete remission [40]. Data reported in literature so far suggest that tumoral melanosis may predict a positive response to treatment in patients with advanced melanoma on immunotherapy [34,39,40,42,43]. ICI therapies upregulating cytotoxic T cell activity may increase tumor antigen detection and scavenger macrophage influx. Hypothetically, these events would enhance melanoma destruction and the concomitant observed tumoral melanosis [39]. However, few patients also showed disease progression in spite of the development of tumoral melanosis during immunotherapy [35,43].

The early onset of disseminate melanosis cutis on photoexposed areas during treatment with pembrolizumab has also been reported in two patients with rapid metastatic melanoma disease progression and failure, suggesting that this condition could be a predictor of negative response to anti-PD-1 therapy [44].

The amount of tumoral melanosis cases is nowadays still limited, and therefore it is difficult to make extensive conclusions on prognosis. Melanosis must always be interpreted in clinical context and considering that these lesions could resemble cutaneous metastases, it is recommended to perform a diagnostic skin biopsy before any therapeutic decisions in melanoma patients.

## 5. Hair and Nail Toxicities

**Figure 3:**
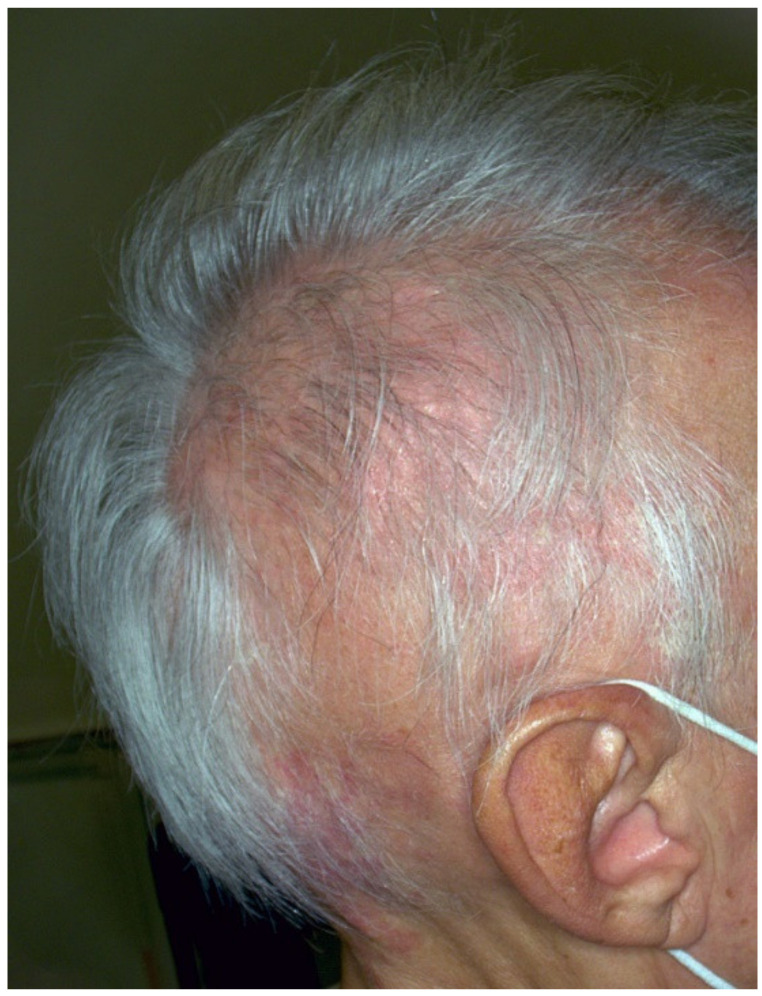
Diffuse patchy alopecia areata ICIs related on right parieto-occipital area.

Hair toxicities related to ICIs are rapidly growing in the literature. Pigmentation disorders of the hair related to immunotherapy during advance melanoma treatment can be classified as generalized, in the course of alopecia areata (AA) (Figure 3) or as localized events.

Alopecia areata is an autoimmune non-cicatricial alopecia, with an unknown etiology. However, the leading pathogenetic hypothesis is that a triggering element, still unclear, generates the autoimmune process in genetically predisposed subjects. The main factors implicated in its development are environmental such as infections or toxins, immunologic, including positive personal of familiar anamnestic record for autoimmune diseases, and genetic. Histologically, a peribulbar CD4+ and CD8+ T lymphocytic infiltrate around anagen phase hair follicles is observed in AA as part of the autoimmune process against hair own antigens [45].

A systemic review of the literature included AA in 2% of the patients undergoing nivolumab therapy (10/502) and induced hair color changes were observed in 1.1% of the patients, whereas for pembrolizumab, alopecia and hair changes were reported in 0.9% of patients (5/555) [10]. These events were classified as much lower in frequency than rash, pruritus and vitiligo.

According to Wang et al. [46], PD-L1 is expressed on the hair follicle dermal sheath cup cells, explaining why PD-1 inhibitors may directly cause alopecia.

As a matter of fact, an estimated 1–2% of patients develop patchy alopecia areata or alopecia universalis during ICIs treatment [47]. Hair depigmentation is usually observed in both conditions.

Few cases of AA related to ICIs have been published and little is known about its mechanism and its clinical, dermoscopic and histopathologic features. Recently, Dimitriou et al. [48] have described the case of a 62-year-old woman with stage IIID melanoma who presented with hair depigmentation and patchy alopecia after being treated with anti-PD1, BRAF and MEK inhibitors, and with complete tumoral remission. Dermoscopy showed yellow dots, vellus hair and broken hair, and, after a scalp biopsy, supported a diagnosis of immune-related AA (ir-AA). She was then treated with topical steroid with regrowth of white hair.

Localized hair depigmentation can be described as an example of antigenic immunity in melanoma patients, where hair loss results as the consequence of this “target out” attack. It has been postulated that T-lymphocyte-mediated cross reactivity with predominant intrafollicular infiltrates of CD8+ against antigens shared between tumor cells and melanocytes on previously immune privileged area on the hair follicle may be an indicator of response in patients treated with therapy by ICI.

Amini Adle et al. [49] have described a case of unilateral poliosis of the right eyebrow and eyelashes, together with a halo of depigmentation around all skin metastases, during treatment with nivolumab, but the asymmetrical distribution was not explained. Recently, a case of bilateral eyelashes poliosis as first sign of undiagnosed metastatic melanoma was described [50].

Another interesting case report [51] describes a 52-year-old woman presenting with cutaneous stage IIIC melanoma of the right eyelid which developed eyelash poliosis within 2 months of combined immunotherapy with ipilimumab and nivolumab that persisted for 3 years of follow-up. In the literature, it has been proven that this rare AE correlates with the regression of malignant melanoma lesions, and thus it reflects treatment efficacy.

Pigmentation effects on nail are very rare and data are lacking about this topic.

A generalized bluish-gray nail discoloration induced by nivolumab therapy for a relapsing melanoma has been reported in a recent paper [52]. This phenomenon gradually expanded over the course of 10 days. No nail pain nor friability was appreciated. The patient denied any changes to his medications or assumption of new drugs since the initiation of nivolumab. This effect regressed after the nivolumab withdrawal.

We believe that the paucity of reports of hair and nail pigmentary changes during immunotherapy for advance melanoma treatment might also be due to an underestimation of these adverse events.
